# Adaptation and Fatigue Model for Neuron Networks and Large Time Asymptotics in a Nonlinear Fragmentation Equation

**DOI:** 10.1186/2190-8567-4-14

**Published:** 2014-07-24

**Authors:** Khashayar Pakdaman, Benoît Perthame, Delphine Salort

**Affiliations:** 1 Institut Jacques Monod, UMR 7592, Université Paris Diderot, 75205, Paris, France; 2 Laboratoire Jacques-Louis Lions, UMR 7598, UPMC Université Paris 06, Sorbonne Universités, 75005, Paris, France; 3 INRIA-Paris-Rocquencourt EPI BANG, Paris, France; 4 Institut Universitaire de France, Paris, France

**Keywords:** Neural networks, Fragmentation equation, Desynchronization, Large time asymptotics

## Abstract

Motivated by a model for neural networks with adaptation and fatigue, we study a conservative fragmentation equation that describes the density probability of neurons with an elapsed time *s* after its last discharge.

In the linear setting, we extend an argument by Laurençot and Perthame to prove exponential decay to the steady state. This extension allows us to handle coefficients that have a large variation rather than constant coefficients. In another extension of the argument, we treat a weakly nonlinear case and prove total desynchronization in the network. For greater nonlinearities, we present a numerical study of the impact of the fragmentation term on the appearance of synchronization of neurons in the network using two “extreme” cases.

**Mathematics Subject Classification (2000)2010:** 35B40, 35F20, 35R09, 92B20.

## 1 Introduction

This article is devoted to study the large time behavior of the solution to a conservative aggregation-fragmentation equation, a class of equations that arises in many applications and that has been widely studied both in the linear case [8,14,18,19] and with nonlinearities [6,9-11,13,20]. 

Our particular motivation is an extension of the elapsed time neural population model, a partial differential equation structured by “age” studied in [15-17], and which gives a new approach to the understanding of synchronization/desynchronization of neural assemblies with respect to the strength of their interconnections. Here, we add a fragmentation term in the model in order to incorporate the fact that the dynamics of the neurons are also related to their past activity, notably that neurons display adaptation and fatigue. That is a progressive decrease of their propensity to firing in response to a step maintained current. This is one of the most common neuronal properties that can introduce correlation in firing times. In this work, we examine whether and how the inclusion of this property can affect the dynamics of neural assemblies. As a consequence, the mathematical study of this equation is more complex. Based on the ideas in [12], we give a new result of exponential decay of the solution to its stationary state in the case where the network is weakly connected. 

We consider the following equation:

(1) $$\left\{ \begin{array}{c} \frac{\partial n(s,t)}{\partial t}+\frac{\partial n(s,t)}{\partial s}+p(s,N(t))n(s,t)\\ \;=\int K(s,u)p(u,N(t))n(u,t)\;du,\;s\geq 0,t\geq 0,\\ n(0,t)=0,\;\;N(t):=\int_{0}^{+\infty }p(s,N(t))n(s,t)\;ds,\\ n(s,0)=n^{0}(s)\geq 0,\;\int_{0}^{\infty }n^{0}(s)\;ds=1. \end{array}\right.$$

• n(s,t) denotes the probability density of neurons at time *t* such that the time elapsed since the last discharge is *s*. It is a fundamental property which follows from our assumptions that, for all times t≥0,

(2) $$\int_{0}^{+\infty }n(s,t)\;ds=\int_{0}^{+\infty }n^{0}(s)\;ds=1,\;n(s,t)\geq 0.$$

• N(t) represents the flux of neurons which discharge at time *t* and is identified to the global amplitude of stimulation of the network.

• p(s,N) models the firing rate of neurons submitted to a stimulation of amplitude *N* and such that the time elapsed since the last discharge is *s*. The coupling between the neurons is taking into account via the function *p* which varies according to the global activity N(t). Hence, in this model, the strength of interconnections between the neurons is taking into account via the variations of *p* with respect to the variable *N*.

• The kernel K(s,u)∈M([0,∞)×[0,∞)), the set of nonnegative measures in [0,∞)×[0,∞), gives the distribution of neurons which take the state *s* when a discharge occurs after an elapsed time *u* since their last discharge.

The structured nature of Eq. (1) is related to the choice of the description of the dynamic of the neurons, which is made via the time elapsed since their last discharge. The term “fragmentation” stems from the fact that, at each time, the density of neurons which discharge is fragmented, via *K*, with respect to the new state of neurons after their discharge; each fragment is given by the flux of neurons which discharge and come back in a same state *s*.

The main question we address here is to prove exponential convergence as t→∞ for the nonlinear problem (1) to a steady state solution A(s), that is,

(3) $$\left\{ \begin{array}{c} \frac{\partial A(s)}{\partial s}+p(s,A^{*})A(s)=\int K(s,u)p(u,A^{*})A(u)\;du,\;s\geq 0,\\ A(0)=0,\;\;\int_{0}^{\infty }A(s)\;ds=1,\;\;A^{*}=\int p(s,A^{*})A(s)\;ds. \end{array}\right.$$

The existence of a stationary solution is proved in Sect. 5 and we attack to convergence through an adaptation of the strategy in [16]. For the linear problem, we construct some kind of spectral gap which opens the door to also treat ‘small’ (in a weak sense) nonlinearities. 

The paper is organized as follows. In Sect. 2, we state our main results after giving assumptions on the coefficients; we separate the linear and nonlinear cases because we can prove much stronger results in the linear case. In Sect. 3, we study the solution of the linear version of Eq. (1) more precisely we prove its large time convergence to the stationary state with exponential decay; this is the proof of Theorem 2.1. Section 4 is devoted to the nonlinear case and to the proofs of Theorems 2.2 and 2.3. We prove the existence of stationary states, i.e., solutions to (3) in Sect. 5. In the last Sect. 6, we present numerical results in the case where the nonlinearity is strong enough to obtain periodic solutions to understand the effect of the fragmentation term in regard to the appearance of spontaneous activity in the network. Several general or technical results are postponed to Appendices in order to focus more the proofs on the main arguments.

## 2 Assumptions and Main Results

We need technical assumptions on the coefficients *p* and *K* in (1) and before we write them in full generality, we begin with a particular example. For the kernel *K*, a Dirac mass at 0, $K(s,u):=\delta_{s=0}$, the equation is equivalently written as a age structured equation and this situation is covered in [16,17]. In this case, the interpretation is clear: After they discharge, all neurons take the same state s=0, irrespective of the time elapsed since their last discharge.

A more general example of kernel *K* is to take

K(s,u):=δ(s−ψ(u)),

where *ψ* is a given increasing function. In this situation, the post discharge state *s* of the neurons only depends on their discharge state *u* (a cumulative time elapsed since their last discharge). Still more general is when K(s,⋅) is a function: this includes variability in the neuron population or randomness in their behavior.

Also, a convenient example of discharge rate p(s,⋅) is a regularized Heavyside function

$$p(s,N)=H_{\delta }\left(s-\sigma (N)\right),\;0<\sigma_{-}\leq \sigma (\cdot )\leq \sigma_{+}<\infty ,$$

with *σ* a given smooth function. It is a caricature for modeling three desirable properties of the neurons: 

• immediately after a discharge, the neuron enters a refractory period, i.e., after a discharge, a neuron cannot discharge again during a certain time interval; this is the assumption that p(s,N)=0 for *s* small,

• after the end of its refractory period, the neuron rapidly recovers a significative sensitive state,

• for an excitatory system, a larger stimulation on the neuron induces smaller refractory period that $\frac{\partial }{\partial N}p(s,N)>0$ and this is written here as $\sigma^{\prime }<0$ even though this assumption is not used in the present analysis.

Those examples of functions *p* and *K* are covered by more general assumptions, and links between these quantities, which we explain now. In Appendix A, we give explicitly the conditions on the two functions *σ* and *ψ* which are induced by our assumptions below.

### 2.1 General Assumptions

#### Assumptions on the Rate p(s,N)

(4) $$0\leq p(s,N)\leq p_{M}<+\infty ,\;\int_{0}^{\infty }\left|\frac{\partial }{\partial s}p(s,N)\right|\;ds<+\infty .$$

There is a bounded function $\sigma :(0,\infty )\to (0,\sigma_{+}]$ such that

(5) $$p(s,N)\equiv p_{M}\;\forall s\geq \sigma (N),\;\;p(s,N)<p_{M}\;\text{for }s<\sigma (N).$$

Finally, we assume

(6) $$\begin{array}{rcl} & & {∥\frac{\partial }{\partial s}p∥}_{L^{\infty }([0,\infty )\times [0,\infty ))}+{∥\frac{\partial^{2}}{\partial s^{2}}p∥}_{L^{\infty }([0,\infty )\times [0,\infty ))}\\ & & \;+{∥\partial_{s}\partial_{N}p∥}_{L^{\infty }([0,\infty )\times [0,\infty ))}<+\infty , \end{array}$$

(7) $${∥\frac{\partial }{\partial N}p∥}_{L^{\infty }([0,\infty )\times [0,\infty ))}\leq \eta <1\;\text{and small enough}.$$

In particular, this assumption and (5) guarantee that, for *n* a probability density, there is a unique positive solution *N* to

$$N=\int_{0}^{\infty }p(s,N)n(s)\;ds.$$

In other words, the nonlinearity in (1) is well determined.

#### Assumptions on the Distribution K(s,u)

The first assumption expresses that neurons which discharge in a state *u* come back in an “earlier” state *s*

(8) $$K(s,u)\geq 0,\;\;K(s,u)=0\;\forall s>u,\;\;\int_{s=0}^{u}K(s,u)\;ds=1\;\forall u>0.$$

These assumptions are fundamental in order to guarantee that n(⋅,t) is a probability as written in (2), but also that ∫K(s,u)p(u)n(u)du is well defined for *n* an integrable function.

Our second assumption is a structural property on K(⋅,⋅) which appears for aggregation-fragmentation equations in [12]. In our context, it says that the shortest is the time elapsed for a discharge, the earliest is the state after discharge. To do this, we define

(9) $$f(s,u):=\int_{x=0}^{s}K(x,u)\;dx,$$

and assume that

(10) $$\Phi (s,u):=-\frac{\partial }{\partial u}f(s,u)\geq 0.$$

The assumptions (8) imply the following properties:

(11) 0≤f≤1,f(s,u)=1for s≥u,Φ(s,u)=0for s>u.

The third assumption imposes a significant change of state after discharge; namely we assume that there is a constant 0≤θ<1 such that for all u≥0,

(12) $$\int_{0}^{+\infty }\Phi (s,u)\;ds=\frac{\partial }{\partial u}\int_{0}^{u}sK(s,u)\;ds\leq \theta <1.$$

As a consequence,

(13) $$\int_{0}^{u}sK(s,u)\;ds\leq \theta u.$$

#### Assumptions Linking *p* and *K*

The following assumptions which link *p* and *K* allow us to prove, in the case of weakly connected neurons, convergence of the solution of Eq. (1) to the stationary state A(s) in (3) with an exponential rate.

We use the notation

(14) $$p_{*}(s)=p\left(s,A^{*}\right),\;\;\sigma^{*}=\sigma \left(A^{*}\right).$$

Our strongest assumption is a smallness assumption on $\sigma^{*}$ and *θ*, but not on $p_{M}$, which is written as follows. Let

$$B_{*}:=e^{\int_{0}^{\sigma^{*}}p_{*}(w)\;dw}\left[\sigma^{*}\int_{0}^{\sigma^{*}}\left|p_{*}^{\prime }(s)\right|\;ds+\theta \int_{0}^{\sigma^{*}}p_{*}(s)\;ds\right]>0.$$

We assume that

(15) $$B_{*}<1,\;\frac{e^{\int_{0}^{\sigma_{*}}p(w)\;dw}}{1-B_{*}}\int_{0}^{\sigma_{*}}\Phi (u,s)\;du+\int_{\sigma_{*}}^{+\infty }\Phi (u,s)\;du<p_{M}.$$

Notice that the second condition can be replaced by the stronger, but more direct inequality

$$\theta \frac{e^{\int_{0}^{\sigma_{*}}p(w)\;dw}}{1-B_{*}}<p_{M}.$$

In the linear case, we also use sometimes one further assumption

(16) $$\exists \mu >0\;\text{such that}\;\underset{u\leq \sigma }{\sup}p(u)\left[\int_{0}^{u}K(s,u)e^{\mu (u-s)}\;ds-1\right]<\mu .$$

We can precise a little the meaning of this assumption. At μ=0, and reversing (13) with some $0\leq \theta_{-}<\theta$, we compute

$$\frac{d}{d\mu }\left[\int_{0}^{u}K(s,u)e^{\mu (u-s)}\;ds-1\right]=\int_{0}^{u}K(s,u)e^{\mu (u-s)}(u-s)\;ds∼u(1-\theta_{-})$$

so that (16) holds for *μ* small if

$$(1-\theta_{-})\underset{0\leq u\leq \sigma }{\sup}p(u)u<1.$$

This is again to say that *σ* is small enough but not necessarily $p_{M}$.

### 2.2 Exponential Decay for the Linear Equation

The linear equation arises as the limiting case where we neglect interconnections within the network, that is,

p(s,N)≡p(s),σ(N)≡σ.

Our first theorem gives a result of exponential decay of solutions to the linear equation of (1) toward the steady state *A* built in Sect. 5. There are several routes toward this goal. A spectral gap can be proved using Poincaré type inequalities; this idea has been developed in [1,4,5] and is for smooth kernels *K*. A probabilistic approach has also been developed; see [2,3] and the references therein. 

Here, we follow yet another approach, developed in [12,19], which handles singular kernels as measures and Dirac masses. It uses the auxiliary functions

(17) m(s,t):=n(s,t)−A(s),

(18) $$M(s,t)=\int_{0}^{s}\left[n(x,t)-A(x)\right]\;dx=\int_{0}^{s}m(x,t)\;dx,$$

(19) $$J(s,t)=\frac{\partial }{\partial t}M(s,t).$$

With these notations and the function P(s)>0 (with $p_{*}=p$ in the linear case at hand) constructed in Appendix C, we can state our first result.

**Theorem 2.1** (Exponential decay. Linear case)

*We make the assumptions* (4), (5), (8), (10), (12), (15), (16) *onpandKwithpindependent on the variableN*, *and assume that*

(20) ∫P(s)|M(s,0)|ds<+∞,∫P(s)|J(s,0)|ds<+∞,

*where*P(s)*is a function uniformly bounded from below defined in Lemma *C.1. *Then there exists*ν>0*such that*

$$\begin{array}{rcl} & & \int_{0}^{\infty }P(s)\left|n(s,t)-A(s)\right|\;ds\\ & & \;\leq Ce^{-\nu t}(\int_{0}^{+\infty }P(s)\left|M(s,0)\right|\;ds+\int_{0}^{+\infty }P(s)\left|J(s,0)\right|\;ds\\ & & \;\;+\int_{0}^{\sigma }\left|n(s,0)-A(s)\right|\;ds). \end{array}$$

### 2.3 Exponential Decay for the Nonlinear Equation

With the notations and preparation of the linear case, we can state our second theorem on exponential decay for weakly nonlinear equation and when *p* is smooth enough. For a better presentation of the proofs, we separate our statements in two theorems.

**Theorem 2.2** (Exponential decay. Nonlinear case)

*We make all the assumptions of Sect*. 2.1 *onpandK*, *and assume furthermore that*

∫P(s)|M(s,0)|ds<+∞,

*still withPconstructed in Lemma *C.1. *Then there exists*C>0, ν>0*such that*

(21) $$\int_{0}^{\infty }P(s)\left|M(s,t)\right|\;ds\leq e^{-\nu t}\int_{0}^{+\infty }P(s)\left|M(s,0)\right|\;ds,$$

(22) $$\left|A^{*}-N(t)\right|\leq Ce^{-\nu t},$$

(23) $$\left|N^{\prime }(t)\right|\leq Ce^{-\nu t}.$$

**Theorem 2.3** (Decay on *m*)

*With the assumptions of Theorem *2.2, *with*

∫P(s)|J(s,0)|ds<+∞,

*there exist two constants*C>0, ν>0*such that*

(24) $$\int_{0}^{+\infty }\left|m(s,t)\right|\;ds\leq Ce^{-\nu t}.$$

## 3 Exponential Decay for the Linear Equation

We prove the time decay with exponential rate as stated in Theorem 2.1, for the linear equation. In this situation, the function *p* does not depend on *N* and assumption (5) can be written as

$$p(s,N)\equiv p(s),\;\;p(s)=p_{M}\;\forall s>\sigma .$$

The strategy of the proof of Theorem 2.1 is to observe that exponential decay for |n(s,t)−A(s)| follows from exponential decay for the functions M(s,t) and its first time derivative, which is much easier to prove than for *m* itself; the counterpart is that it involves a weighted norm, as expressed in Theorem 2.1, at variance with the Poincaré method in [1,4,5]. Indeed, there are two main advantages of considering the solutions M(s,t), J(s,t), instead of m(s,t):=n(s,t)−A(s); (i) they satisfy a closed equation, (ii) the dual problem to the corresponding stationary equation has a negative first eigenvalue. This directly implies exponential decay of both $\int_{0}^{+\infty }P(s)|M(s,t)|\;ds$ and $\int_{0}^{+\infty }P(s)|J(s,t)|\;ds$.

We split the proof of Theorem 2.1 in two steps: 

• In the first part, we check that the proof of Theorem 2.1 is a direct consequence of the exponential decay in $L^{1}$ of P(s)|M(s,t)| and P(s)|J(s,t)|.

• The second part is devoted to prove exponential decay in $L^{1}$ of P(s)|M(s,t)| and P(s)|J(s,t)|.

### 3.1 Reduction to Exponential Decay on M(s,t) and $\frac{\partial }{\partial t}M(s,t)$

We derive the Theorem 2.1 from the following proposition.

**Proposition 3.1***Assume that there are constants*λ<0, B>0*and a functionPsuch that*

(25) $$\frac{1}{B}\leq P(x)\leq BP(y)\;\text{for }0\leq x\leq y,$$

and

$$\left\{ \begin{array}{c} \int_{0}^{+\infty }P(s)|M(s,t)|\;ds\leq Ce^{\lambda t}\int_{0}^{+\infty }P(s)|M(s,0)|\;ds,\\ \int_{0}^{+\infty }P(s)|J(s,t)|\;ds\leq Ce^{\lambda t}\int_{0}^{+\infty }P(s)|J(s,0)|\;ds. \end{array}\right.$$

*Then there exists a constantCand a*ν>0*such that*

$$\begin{array}{rcl} & & \int_{0}^{+\infty }\left|n(s,t)-A(s)\right|\;ds\\ & & \;\leq Ce^{-\nu t}(\int_{0}^{+\infty }P(s)\left|M(s,0)\right|\;ds+\int_{0}^{+\infty }P(s)\left|J(s,0)\right|\;ds\\ & & \;\;+\int_{0}^{\sigma }\left|n(s,0)-A(s)\right|\;ds). \end{array}$$

*Proof* Using Eq. (29) for *M* (see the first step of the proof of Proposition 3.2), we obtain

(26) $$\begin{array}{rcl} m(s,t) & = & \frac{\partial }{\partial s}M(s,t)\\ & = & -J(s,t)-p(s)M(s,t)\\ & & -\int_{u=s}^{+\infty }p^{\prime }(u)M(u,t)f(s,u)\;du+\int \Phi (s,u)p(u)M(u,t)\;du. \end{array}$$

We first use this relation to handle the values s>σ; then $p^{\prime }(u)=0$ in (26) and it is reduced to

|m(s,t)|≤|J(s,t)|+|p(s)M(s,t)|+∫Φ(s,u)p(u)|M(u,t)|du.

Multiplying this inequality by *P*, integrating between *σ* and +∞ with respect to the variable *s*, we obtain using estimate (11) and that Φ(s,u)=0 for s>u, that

$$\begin{array}{rcl} \int_{\sigma }^{+\infty }P(s)\left|m(s,t)\right|\;ds & \leq & 2\int_{\sigma }^{+\infty }P(s)\left(\left|M(s,t)\right|+\left|J(s,t)\right|\right)\;ds\\ & & +\int_{\sigma }^{+\infty }\left(\int_{\sigma }^{+\infty }\Phi (s,u)P(s)\;ds\right)p(u)\left|M(u,t)\right|\;du. \end{array}$$

Using assumption (12) on *Φ*, we obtain that for all u∈(σ,+∞), there exists $r_{u}\in (\sigma ,u)$ such that

$$\int_{\sigma }^{+\infty }P(s)I_{s<u}\Phi (s,u)\;ds\leq \theta P(r_{u})\leq \theta BP(u),$$

the second inequality being a consequence of assumption (25) on *P*. We deduce that there exists a constant *C* such that

(27) $$\int_{\sigma }^{\infty }P(s)\left|m(s,t)\right|\;ds\leq C\int_{0}^{+\infty }P(s)\left(\left|M(s,t)\right|+\left|J(s,t)\right|\right)\;ds.$$

Finally, with the exponential decay assumptions on *M* and *J* in Proposition 3.1, we conclude that

(28) $$\int_{\sigma }^{\infty }P(s)\left|m(s,t)\right|\;ds\leq Ce^{-\nu t}\int_{0}^{+\infty }P(s)\left(\left|M(s,0)\right|+\left|J(s,0)\right|\right)\;ds.$$

Next, we control the small values of *s*, namely $\int_{0}^{\sigma }|m(s,t)|\;ds$. We cannot control this quantity directly and proceed to estimate $\int_{0}^{\sigma }e^{-\mu s}|m(s,t)|\;ds$ for *μ* given in condition (16).

We set $v(s,t):=e^{-\mu s}m(s,t)$. Then, for s∈[0,σ], *v* satisfies the equation

$$\frac{\partial }{\partial t}v+\frac{\partial }{\partial s}v+(\mu +p)v=\int_{0}^{+\infty }p(u)K(s,u)e^{-\mu s}m(u,t)\;du$$

and thus

$$\frac{\partial }{\partial t}|v|+\frac{\partial }{\partial s}|v|+(\mu +p)|v|\leq \int_{0}^{+\infty }p(u)K(s,u)e^{-\mu s}\left|m(u,t)\right|\;du.$$

Integrating the above equation, using that m(0,t)=0 and (8), we obtain

$$\begin{array}{rcl} & & \frac{d}{dt}\int_{0}^{\sigma }\left|v(t,s)\right|\;ds+\mu \int_{0}^{\sigma }\left|v(t,s)\right|\;ds\\ & & \;\leq \int_{0}^{\sigma }\int_{0}^{+\infty }p(u)K(s,u)e^{-\mu s}\left|m(u,t)\right|\;du\;ds-\int_{0}^{\sigma }p(u)\left|v(u,t)\right|\;du\\ & & \;\leq \int_{0}^{\sigma }p(u)\left(\int_{0}^{u}K(s,u)e^{\mu (u-s)}\;ds-1\right)\left|v(u,t)\right|\;du+p_{M}\int_{\sigma }^{+\infty }\left|m(t,u)\right|\;du. \end{array}$$

Therefore, using assumption (16) and estimate (28), there is a ν>0 such that

$$\frac{d}{dt}\int_{0}^{\sigma }\left|v(t,s)\right|\;ds\leq -\nu \int_{0}^{\sigma }\left|v(t,s)\right|\;ds+Ce^{\lambda t}\int_{0}^{\infty }P(s)\left(\left|M(s,0)\right|+\left|J(s,0)\right|\right)\;ds.$$

This concludes the proof of Proposition 3.1. □

### 3.2 Exponential Decay of *M* and $\frac{\partial }{\partial t}M$

We now establish the assumptions in Proposition 3.1 on exponential decay for *M* and *J*. This is stated in the following proposition.

**Proposition 3.2***There exist a constant*λ<0*and a functionPsatisfying properties* (25) *such that the following estimates hold*

$$\begin{array}{rcl} \int_{0}^{+\infty }P(s)\left|M(s,t)\right|\;ds & \leq & Ce^{\lambda t}\int_{0}^{+\infty }P(s)\left|M(s,0)\right|\;ds,\\ \int_{0}^{+\infty }P(s)\left|J(s,t)\right|\;ds & \leq & Ce^{\lambda t}\int_{0}^{+\infty }P(s)\left|J(s,0)\right|\;ds, \end{array}$$

*assuming the initial bounds* (20) *are satisfied*.

*Proof* We divide the proof in two steps. We first derive a closed form for the equation on M(s,t); this is our main observation, which allows to extend the argument in [12] to nonconstant coefficients. Then, thanks to dual problem that we study in Appendix C, we conclude our proof.

**Step 1. The equation on**M(s,t)**.** Integrating Eq. (1) once subtracted to the same equation for *A*, we find successively

$$\begin{array}{rcl} & & \frac{\partial M(s,t)}{\partial t}+\frac{\partial M(s,t)}{\partial s}+\int_{0}^{s}p(x)\frac{\partial M(x,t)}{\partial x}\;dx\\ & & \;=\int_{x=0}^{s}\int K(x,u)p(u)\frac{\partial M(u,t)}{\partial u}\;du\;dx,\\ & & \frac{\partial M(s,t)}{\partial t}+\frac{\partial M(s,t)}{\partial s}+p(s)M(s,t)\\ & & \;=\int_{0}^{s}\frac{\partial p(x)}{\partial x}M(x,t)\;dx-\int_{x=0}^{s}\int \frac{\partial }{\partial u}\left(K(x,u)p(u)\right)M(u,t)\;du\;dx,\\ & & \frac{\partial M(s,t)}{\partial t}+\frac{\partial M(s,t)}{\partial s}+p(s)M(s,t)\\ & & \;=\int_{u=0}^{s}\frac{\partial p(u)}{\partial u}M(u,t)\;du-\int_{u=0}^{\infty }\frac{\partial p(u)}{\partial u}M(u,t)\int_{x=0}^{s}K(x,u)\;dx\;du\\ & & \;\;-\int_{x=0}^{s}\int \frac{\partial K(x,u)}{\partial u}p(u)M(u,t)\;du\;dx,\\ & & \frac{\partial M(s,t)}{\partial t}+\frac{\partial M(s,t)}{\partial s}+p(s)M(s,t)\\ & & \;=\int_{u=0}^{s}\frac{\partial p(u)}{\partial u}\left(1-f(s,u)\right)M(u,t)\;du-\int_{u=s}^{\infty }\frac{\partial p(u)}{\partial u}f(s,u)M(u,t)\;du\\ & & \;\;+\int \Phi (s,u)p(u)M(u,t)\;du. \end{array}$$

As f(s,u)=1 for s≥u, this equality reduces to

(29) $$\begin{array}{rcl} & & \frac{\partial M(s,t)}{\partial t}+\frac{\partial M(s,t)}{\partial s}+p(s)M(s,t)\\ & & \;=-\int_{u=s}^{\infty }\frac{\partial p(u)}{\partial u}f(s,u)M(u,t)\;du+\int p(u)\Phi (s,u)M(u,t)\;du. \end{array}$$

We can now insert the absolute values and find

(30) $$\begin{array}{rcl} & & \frac{\partial |M(s,t)|}{\partial t}+\frac{\partial |M(s,t)|}{\partial s}+p(s)\left|M(s,t)\right|\\ & & \;\leq \int_{u=s}^{\infty }\left|p^{\prime }(u)\right|f(s,u)\left|M(u,t)\right|\;du+\int p(u)\Phi (s,u)\left|M(u,t)\right|\;du. \end{array}$$

There are to routes to go further. To cover the case of interest where *p* can vanish during the refractory period, we use a duality argument. However, we can use directly formula (30) under different assumptions on *p*; this is performed in Appendix B.

**Step 2. End of the proof of Proposition 3.2.** By construction, we have M(0,t)=M(∞,t)=0. Therefore, we may multiply inequality (30) by *P*, using Lemma C.1 and assumption (20), we may integrate by parts and we find that there exists λ<0 such that

(31) $$\frac{d}{dt}\int \left|M(s,t)\right|P(s)\;ds\leq \lambda \int \left|M(s,t)\right|P(s)\;ds,$$

which proves the decay of |M| in Proposition 3.2 thanks to the Gronwall lemma.

Because time only enters in Eq. (29) through M(s,t), we may differentiate in time and find that *J* still satisfies (29), therefore, the inequality (30) also holds for |J| and, since 0=J(0,t)=J(∞,t), we conclude as before that

$$\frac{d}{dt}\int \left|J(s,t)\right|P(s)\;ds\leq \lambda \int \left|J(s,t)\right|P(s)\;ds$$

which concludes the proof of Proposition 3.2. □

## 4 Exponential Decay for the Nonlinear Case

The proofs of Theorems 2.2 and 2.3 follow the strategy used to prove Theorem 2.1. The main difficulty is that the control on *M* is a weak control on *m* while the nonlinear term, which involves N(t), is in a strong dependency. To solve this difficulty, we had to assume that the function *p* is regular enough; this allows us, via an integration by parts to increase the regularity of the nonlinear term N(t) at the expense of Lipschitz regularity on *p*.

**Step 1. Proof of (21) and (22).** Let *n* be the solution of Eq. (1) and *A* be the stationary state in (3). Then m:=n−A is solution of the equation

$$\left\{ \begin{array}{c} \frac{\partial m(s,t)}{\partial t}+\frac{\partial m(s,t)}{\partial s}+p(s,A^{*})m(s,t)\\ \;=\int K(s,u)p(u,A^{*})m(u,t)\;du\\ \;\;+[p(s,A^{*})-p(s,N(t))]n(s,t)\\ \;\;-\int K(s,u)[p(u,A^{*})-p(u,N(t))]n(u,t)\;du,\;s\geq 0,t\geq 0,\\ m(0,t)=0,\\ m(s,0)=n^{0}(s)-A(s),\;\;\int_{0}^{\infty }m^{0}(s)\;ds=0,\;\;A^{*}:=\int p(s,A^{*})A(s)\;ds. \end{array}\right.$$

Following the calculation in the linear case, the function $M(s,t):=\int_{0}^{s}m(u,t)\;du$ is solution of the equation:

(32) $$\left\{ \begin{array}{c} \frac{\partial M(s,t)}{\partial t}+\frac{\partial M(s,t)}{\partial s}+p(s,A^{*})M(s,t)\\ \;=-\int_{u=s}^{\infty }\frac{\partial p(u,A^{*})}{\partial u}f(s,u)M(u,t)\;du+\int \Phi (s,u)p(u,A^{*})M(u,t)\;du\\ \;\;+\int_{0}^{s}[p(u,A^{*})-p(u,N(t))]n(u,t)\;du\\ \;\;-\int f(s,u)[p(u,A^{*})-p(u,N(t))]n(u,t)\;du. \end{array}\right.$$

We introduce the remainder term

$$\begin{array}{rcl} R(s,t) & := & \int_{0}^{s}\left[p\left(u,A^{*}\right)-p\left(u,N(t)\right)\right]n(u,t)\;du\\ & & -\int f(s,u)\left[p\left(u,A^{*}\right)-p\left(u,N(t)\right)\right]n(u,t)\;du \end{array}$$

which can be written using (11) as

$$R(s,t)=-\int_{s}^{+\infty }f(s,u)\left[p\left(u,A^{*}\right)-p\left(u,N(t)\right)\right]n(u,t)\;du.$$

Using assumptions (7) and (5), we find that

(33) $$\left|R(s,t)\right|\leq \{\begin{array}{cc} 0 & \text{for }s\geq \sigma_{+}:=\max\sigma (\cdot ),\\ 2\eta |A^{*}-N(t)| & \text{else.} \end{array}$$

As before, we may enter the absolute values and find

$$\begin{array}{rcl} & & \frac{\partial |M(s,t)|}{\partial t}+\frac{\partial |M(s,t)|}{\partial s}+p\left(s,A^{*}\right)\left|M(s,t)\right|\\ & & \;\leq \int_{u=s}^{\infty }\left[|\frac{\partial p(u,A^{*})}{\partial u}|f(s,u)+\Phi (s,u)p\left(u,A^{*}\right)\right]\left|M(u,t)\right|\;du+\left|R(s,t)\right|. \end{array}$$

Multiplying by *P* the equation obtained for *M*, where *P* is as in Lemma C.1, we conclude that there is λ<0 (and close to 0) such that

(34) $$\frac{d}{dt}\int P(s)\left|M(s,t)\right|\;ds\leq \lambda \int P(s)\left|M(s,t)\right|\;ds+2\eta {∥P∥}_{L^{\infty }(0,\sigma_{+})}\left|A^{*}-N(t)\right|.$$

To proceed further, using the notation (17) and M(0,t)=0, we write

$$\begin{array}{rcl} A^{*}-N(t) & = & \int_{0}^{\infty }\left[p\left(s,A^{*}\right)A(s)-p\left(s,N(t)\right)n(s,t)\right]\;ds\\ & = & -\int_{0}^{\infty }p\left(s,A^{*}\right)m(s,t)\;ds+\int_{0}^{\infty }\left[p\left(s,A^{*}\right)-p\left(s,N(t)\right)\right]n(s,t)\;ds\\ & = & \int_{0}^{\infty }\frac{\partial }{\partial s}p\left(s,A^{*}\right)M(s,t)\;ds+\int_{0}^{\infty }\left[p\left(s,A^{*}\right)-p\left(s,N(t)\right)\right]n(s,t)\;ds \end{array}$$

from which we conclude

$$\left|A^{*}-N(t)\right|\leq \frac{1}{\inf_{s\geq 0}P}{∥\partial_{s}p∥}_{L^{\infty }([0,\infty )\times [0,\infty ))}\int P(s)\left|M(s,t)\right|\;ds+\eta \left|N(t)-A^{*}\right|$$

and finally

(35) $$\left|A^{*}-N(t)\right|\leq \frac{1}{(1-\eta )\inf_{s\geq 0}P}{∥\partial_{s}p∥}_{L^{\infty }([0,\infty )\times [0,\infty ))}\int P(s)\left|M(s,t)\right|\;ds.$$

Since η>0 is supposed small enough, we may insert this estimate in (34) and we deduce that there exists ν>0 such that

$$\frac{d}{dt}\int P(s)\left|M(s,t)\right|\;ds\leq -\nu \int P(s)\left|M(s,t)\right|\;ds$$

which proves the inequality (21) using the Gronwall lemma.

Inserting this exponential decay on ∫P(s)|M(s,t)|ds in (35) proves (22).

**Step 2. Proof of (23).** In order to better explain our strategy, we begin with a global Lipschitz estimate on *N* and then prove the exponential decay.

From the definition of *N*, we have

$$N^{\prime }(t)=\int_{0}^{+\infty }N^{\prime }(t)\partial_{N}p\left(s,N(t)\right)n(s,t)\;ds+\int_{0}^{+\infty }p\left(s,N(t)\right)\partial_{t}n(s,t)\;ds.$$

Using (2), and (7), we obtain

(36) $$(1-\eta )\left|N^{\prime }(t)\right|\leq |\int_{0}^{+\infty }p\left(s,N(t)\right)\partial_{t}n(s,t)\;ds|.$$

We first prove a Lipschitz estimate. We write

$$\begin{array}{rcl} & & \int_{0}^{+\infty }p\left(s,N(t)\right)\partial_{t}n(s,t)\;ds\\ & & \;=-\int_{0}^{+\infty }p\left(s,N(t)\right)\partial_{s}n(s,t)\;ds-\int_{0}^{+\infty }p^{2}\left(s,N(t)\right)n(s,t)\;ds\\ & & \;\;+\int_{0}^{+\infty }p\left(s,N(t)\right)\int_{0}^{+\infty }K(s,u)p\left(u,N(t)\right)n(u,t)\;du\;ds. \end{array}$$

Integration by parts implies that

$$\int_{0}^{+\infty }p\left(s,N(t)\right)\partial_{s}n(s,t)\;ds=-\int_{0}^{+\infty }\partial_{s}p\left(s,N(t)\right)n(s,t)\;ds.$$

The Lipschitz bound on *p* and (2) gives the estimate $|N^{\prime }|\leq C$.

We now prove estimate (23) based on inequality (36). We recall the notations $m=n-A=\partial_{s}M(s,t)$ and conclude

$$\begin{array}{rcl} \int_{0}^{+\infty }p\left(s,N(t)\right)\partial_{t}n(s,t)\;ds & = & \int_{0}^{+\infty }p\left(s,N(t)\right)\partial_{t}m(s,t)\;ds\\ & = & -\int_{0}^{+\infty }\partial_{s}p\left(s,N(t)\right)\partial_{t}M(s,t)\;ds. \end{array}$$

Because *M* satisfies the closed equation (32), we deduce that

$$\begin{array}{rcl} & & -\int_{0}^{+\infty }\partial_{s}p\left(s,N(t)\right)\partial_{t}M(s,t)\;ds\\ & & \;=\int_{0}^{+\infty }\partial_{s}p\left(s,N(t)\right)\partial_{s}M(s,t)\;ds+\int_{0}^{+\infty }\partial_{s}p\left(s,N(t)\right)p\left(s,A^{*}\right)M(s,t)\;ds\\ & & \;\;-\int_{0}^{+\infty }\partial_{s}p\left(s,N(t)\right)\int_{u=s}^{\infty }\frac{\partial p(u,A^{*})}{\partial u}f(s,u)M(u,t)\;du\;ds\\ & & \;\;+\int_{0}^{+\infty }\partial_{s}p\left(s,N(t)\right)\int \Phi (s,u)p\left(u,A^{*}\right)M(u,t)\;du\;ds\\ & & \;\;+\int_{0}^{+\infty }\partial_{s}p\left(s,N(t)\right)R(s,t)\;ds. \end{array}$$

To continue, we control each term by quantities as M(s,t) for which we have already proved exponential decay

$$\begin{array}{rcl} \left|\int_{0}^{+\infty }\partial_{s}p\left(s,N(t)\right)\partial_{s}M(s,t)\;ds\right| & = & \left|\int_{0}^{+\infty }\partial_{s}^{2}p\left(s,N(t)\right)M(s,t)\;ds\right|\\ & \leq & C\int_{0}^{+\infty }\left|M(s,t)\right|\;ds,\\ \left|\int_{0}^{+\infty }\partial_{s}p\left(s,N(t)\right)p\left(s,A^{*}\right)M(s,t)\;ds\right| & \leq & C\int_{0}^{+\infty }\left|M(s,t)\right|\;ds. \end{array}$$

Using assumption (5)

$$|\int_{0}^{+\infty }\partial_{s}p\left(s,N(t)\right)\int_{u=s}^{\infty }\frac{\partial p(u,A^{*})}{\partial u}f(s,u)M(u,t)\;du\;ds|\leq C\int_{0}^{+\infty }\left|M(s,t)\right|\;ds$$

because these integrals vanish for $s>\sigma^{+}$ and $u>\sigma^{+}$. Next, assumption (12) gives

$$|\int_{0}^{+\infty }\int \partial_{s}p\left(s,N(t)\right)\Phi (s,u)p\left(u,A^{*}\right)M(u,t)\;du\;ds|\leq C\int_{0}^{+\infty }\left|M(s,t)\right|\;ds.$$

Finally, estimate (33) and Theorem 2.2 imply that there exist *C* and ν>0 such that

$$\int_{0}^{+\infty }\left|R(s,t)\right|\;ds\leq Ce^{-\nu t}.$$

All these terms have exponential decay, and thus, back to inequality (36), this concludes the proof of (23).

The proof of Theorem 2.2 is now complete.  □

**Step 3. Proof Theorem 2.3.** Thanks to (32), the function $\partial_{t}M(s,t):=J(s,t)$ satisfies the equation

(37) $$\begin{array}{rcl} & & \frac{\partial J(s,t)}{\partial t}+\frac{\partial J(s,t)}{\partial s}+p\left(s,A^{*}\right)J(s,t)\\ & & \;=-\int_{u=s}^{\infty }\frac{\partial p(u,A^{*})}{\partial u}f(s,u)J(u,t)\;du\\ & & \;\;+\int \Phi (s,u)p\left(u,A^{*}\right)J(u,t)\;du+\tilde{R}(s,t) \end{array}$$

with

{R˜(s,t)≡0for s≥σ+,R˜(s,t)=−∫sσ+f(s,u)N′(t)∂Np(u,N(t))n(u,t)duR˜(s,t)=−∫sσ+f(s,u)[p(u,A∗)−p(u,N(t))]∂tn(u,t)dufor s≤σ+.

Inserting absolute values in Eq. (37) and multiplying by *P* built in Lemma C.1, we obtain that there exists λ>0 such that

$$\frac{d}{dt}\int P(x)\left|J(x,t)\right|\;dx\leq -\lambda \int P(x)\left|J(x,t)\right|\;dx+\int_{0}^{+\infty }P(x)\left|\tilde{R}(x,t)\right|\;dx.$$

We claim that there exist C>0, ν>0 such that

(38) $$\int_{0}^{+\infty }P(x)\left|\tilde{R}(x,t)\right|\;dx=\int_{0}^{\sigma^{+}}P(x)\left|\tilde{R}(x,t)\right|\;dx\leq Ce^{-\nu t}.$$

To prove it, we proceed in estimating each term of $\tilde{R}$. For $s\leq \sigma^{+}$, we control the first term as

(39) $$\begin{array}{rcl} & & \int_{s}^{\sigma^{+}}P(s)f(s,u)\left|N^{\prime }(t)\partial_{N}p\left(u,N(t)\right)\right|n(u,t)\;du\\ & & \;\leq \left|N^{\prime }(t)\right|{∥\partial_{N}p∥}_{L^{\infty ([0,\infty )\times [0,\infty ))}}{∥P∥}_{L^{\infty }[0,\sigma^{+}]}\\ & & \;\leq Ce^{-\nu t} \end{array}$$

thanks to the exponential decay on $N^{\prime }$ in (23).

The second contribution to $\tilde{R}$, namely

$$\int_{s=0}^{\sigma^{+}}|\int_{s}^{\sigma^{+}}f(s,u)\left[p\left(u,A^{*}\right)-p\left(u,N(t)\right)\right]\partial_{t}n(u,t)\;du|\;ds$$

is longer to estimate. Using the equation on *n*, it is the sum of three integrals

$$\begin{array}{rcl} & & \int_{s=0}^{\sigma^{+}}|\int_{s}^{\sigma^{+}}f(s,u)\left[p\left(u,A^{*}\right)-p\left(u,N(t)\right)\right]\partial_{u}n(u,t)\;du|\;ds\\ & & \;+\int_{s=0}^{\sigma^{+}}|\int_{s}^{\sigma^{+}}f(s,u)\left[p\left(u,A^{*}\right)-p\left(u,N(t)\right)\right]p\left(u,N(t)\right)n(u,t)\;du|\;ds\\ & & \;+\int_{s=0}^{\sigma^{+}}|\int_{s}^{\sigma^{+}}f(s,u)\left[p\left(u,A^{*}\right)-p\left(u,N(t)\right)\right]\\ & & \;\times \int_{0}^{+\infty }K(u,w)p\left(w,N(t)\right)n(w,t)\;dw\;du|\;ds. \end{array}$$

Using assumption (7) and estimate (22), the last two integrals are controlled by

$$C\left|A^{*}-N(t)\right|\leq Ce^{-\nu t}.$$

It remains to estimate the first integral. We integrate by parts and write

$$\begin{array}{rcl} & & \int_{s}^{\sigma^{+}}f(s,u)\left[p\left(u,A^{*}\right)-p\left(u,N(t)\right)\right]\partial_{u}n(u,t)\;du\\ & & \;=-f(s,s)\left[p\left(s,A^{*}\right)-p\left(s,N(t)\right)\right]n(s,t)\\ & & \;\;+\int_{s}^{\sigma^{+}}\Phi (s,u)\left[p\left(u,A^{*}\right)-p\left(u,N(t)\right)\right]n(u,t)\;du\\ & & \;\;-\int_{s}^{\sigma^{+}}f(s,u)\partial_{u}\left[p\left(u,A^{*}\right)-p\left(u,N(t)\right)\right]n(u,t)\;du. \end{array}$$

Integrating the above equality between 0 and $\sigma^{+}$ and using assumptions (11), (12), (6), (4), (7), we obtain that there exists a constant *C* such that

$$\begin{array}{rcl} & & \int_{s=0}^{\sigma^{+}}|\int_{s}^{\sigma^{+}}f(s,u)\left[p\left(u,A^{*}\right)-p\left(u,N(t)\right)\right]\partial_{u}n(u,t)\;du|\;ds\\ & & \;\leq C\left|A^{*}-N(t)\right|\leq Ce^{-\nu t} \end{array}$$

and the last inequality is again (22). We deduce that

(40) $$\int_{s=0}^{\sigma^{+}}|\int_{s}^{\sigma^{+}}f(s,u)\left[p\left(u,A^{*}\right)-p\left(u,N(t)\right)\right]\partial_{t}n(u,t)\;du|\;ds\leq Ce^{-\nu t}.$$

Putting the estimates (39) and (40) together, we conclude the proof of estimate (38).

Therefore, we conclude that

$$\frac{d}{dt}\int P(x)\left|J(x,t)\right|\;dx\leq -\lambda \int P(x)\left|J(x,t)\right|\;dx+Ce^{-\nu t}$$

and thus there exists constants *C* and ν>0 such that

$$\int_{0}^{+\infty }P(x)\left|J(t,x)\right|\;dx\leq Ce^{-\nu t}.$$

Using the same computations than in the proof of Proposition 3.1 and using the bound (33) on *R*, we obtain Theorem 2.3. □

## 5 Existence of a Stationary State

This section is devoted to the proof of existence and uniqueness of stationary states for Eq. (1) in the case where the network is weakly connected. We begin with the linear case and then we treat the weakly nonlinear case that is weakly connected networks.

### 5.1 The Linear Case

The following theorem holds.

**Theorem 5.1** (Stationary states. Linear case)

*Assume thatKsatisfies assumptions* (8), (12), *and*

(41) $$0\leq p(s)\leq p_{M},\;\;p(s)\geq p^{*}>0\;\text{for }s\geq s^{*}.$$

Then there exists a unique solution of the equation

(42) $$\left\{ \begin{array}{c} \frac{\partial A(s)}{\partial s}+p(s)A(s)=\int K(s,u)p(u)A(u)\;du,\;s\geq 0,\\ A(0)=0,\;\;\int_{0}^{\infty }A(s)\;ds=1. \end{array}\right.$$

The proof is standard [8,14,18,19] and based on the Krein–Rutman theorem (see [7]). 

*Proof of Theorem 5.1* To justify the computations in this proof, we restrict ourselves to the case when *p* and *K* are continuous, which allows us to use a consequence of the Krein–Rutman theorem as recalled in Theorem D.1; this is not a restriction because the extension to our assumptions in Sect. 1 follows from standard regularization argument and passing to the limit as we do it below.

So, as to ensure positivity and compactness, we introduce two truncation parameters ε>0 small enough and R>0 large enough. According to Theorem D.1, there is $\lambda_{\varepsilon ,R}\in R$ and $A_{\varepsilon ,R}\in C^{1}([0,R])$ such that

(43) $$\left\{ \begin{array}{c} \frac{\partial A_{\varepsilon ,R}(s)}{\partial s}+(p(s)+\lambda_{\varepsilon ,R})A_{\varepsilon ,R}(s)\\ \;=\int_{0}^{R}K(s,u)p(u)A_{\varepsilon ,R}(u)\;du,\;0\leq s\leq R,\\ A_{\varepsilon ,R}(0)=\varepsilon ,\;\;A_{\varepsilon ,R}(s)>0,\;\;\int_{0}^{R}A_{\varepsilon ,R}(s)\;ds=1. \end{array}\right.$$

To prove Theorem 5.1, we need to pass to the limit in Eq. (43) when *ε* and $R^{-1}$ go to 0. To do this, it is enough to prove compactness for the eigenvalues $\lambda_{\varepsilon ,R}$ and convergence for the functions $A_{\varepsilon ,R}$ to a function *A* satisfying properties of Theorem 5.1.

We begin with the following a priori estimates

**Lemma 5.2***For all*ε>0*and*R>0*large enough*, *the following two estimates hold*:

(44) $$\begin{array}{rl} \varepsilon -\frac{2}{R} & \leq \lambda_{\varepsilon ,R}\leq \varepsilon ,\\ (1-\theta )\int_{0}^{R}sA_{\varepsilon ,R}(s)\;ds & \leq \frac{1}{p^{*}}+s^{*}(1-\theta ), \end{array}$$

*whereθis defined in* (12) *and*$s^{*}$, $p^{*}$*are defined in* (41).

*Proof* Integrating Eq. (43) between 0 and *x* with respect to the variable *s*, we obtain that

(45) $$\begin{array}{rcl} \lambda_{\varepsilon ,R}\int_{0}^{x}A_{\varepsilon ,R}(s)\;ds & = & \varepsilon -A_{\varepsilon ,R}(x)\\ & & -\int_{0}^{x}p(u)A_{\varepsilon ,R}(u)\;du+\int_{0}^{R}f(x,u)p(u)A_{\varepsilon ,R}(u)\;du. \end{array}$$

Choosing x=R and thanks to assumption (8), we find the upper bound on $\lambda_{\varepsilon ,R}$

(46) $$\lambda_{\varepsilon ,R}=\varepsilon -A_{\varepsilon ,R}(R)\leq \varepsilon .$$

Next, we multiply Eq. (43) by *s* and integrate between 0 and *R*. We find thanks to (13)

$$\begin{array}{rcl} & & -1+RA_{\varepsilon ,R}(R)+\lambda_{\varepsilon ,R}\int_{0}^{R}sA_{\varepsilon ,R}(s)\;ds\\ & & \;\;+\int_{0}^{R}sp(s)A_{\varepsilon ,R}(s)\;ds\int_{0}^{R}\int_{0}^{R}sK(s,u)p(u)A_{\varepsilon ,R}(u)\;du\\ & & \;\leq \theta \int_{0}^{R}up(u)A_{\varepsilon ,R}(u)\;du, \end{array}$$

which we can also write, using the above expression of $\lambda_{\varepsilon ,R}$, as

$$\begin{array}{rcl} & & (1-\theta )\int_{0}^{R}sp(s)A_{\varepsilon ,R}(s)\;ds+\varepsilon \int_{0}^{R}sA_{\varepsilon ,R}(s)\;ds\\ & & \;\leq 1+A_{\varepsilon ,R}(R)\left[\int_{0}^{R}sA_{\varepsilon ,R}(s)\;ds-R\right]\leq 1. \end{array}$$

Using (41), we conclude that

$$(1-\theta )\int_{0}^{R}sA_{\varepsilon ,R}(s)\;ds\leq \frac{1}{p^{*}}+s^{*}(1-\theta ),$$

which concludes the proof of the second estimate of Lemma 5.2. And this estimate, in the previous identity also gives

$$\left[R-s^{*}-\frac{1}{p^{*}(1-\theta )}\right]A_{\varepsilon ,R}(R)\leq 1.$$

And for *R* large enough, we obtain

$$A_{\varepsilon ,R}(R)\leq \frac{2}{R}.$$

The formula (46) concludes the lower estimate for $\lambda_{\varepsilon ,R}$ and the proof of the Lemma 5.2. □

We continue our a priori estimates with the following lemma.

**Lemma 5.3***For*ε>0*small enough and*R>0*large enough*, *we have*

$${∥A_{\varepsilon ,R}∥}_{L^{\infty }([0,\infty ))}\leq p_{M}+2\varepsilon +\frac{2}{R},\;\;{∥\frac{\partial }{\partial s}A_{\varepsilon ,R}∥}_{L^{1}([0,\infty ))}\leq p_{M}+\varepsilon +\frac{2}{R}.$$

*Proof* From Eq. (45), we have

$$\begin{array}{rcl} A_{\varepsilon ,R}(x) & \leq & \varepsilon -\lambda_{\varepsilon ,R}\int_{0}^{x}A_{\varepsilon ,R}(s)\;ds+\int_{0}^{R}f(x,u)p(u)A_{\varepsilon ,R}(u)\;du\\ & \leq & \varepsilon +|\lambda_{\varepsilon ,R}|+{∥p∥}_{L^{\infty }([0,\infty ))} \end{array}$$

and, from the estimate in Lemma 5.2, we deduce the $L^{\infty }$ bound on $A_{\varepsilon ,R}$ in Lemma 5.3. The other bound follows directly from the equation. □

Using Lemma 5.3, we may extract from $A_{\varepsilon ,R}$ a subsequence which converges locally strongly to a function $A\in L^{\infty }([0,\infty ))$. Moreover, using the first estimate of Lemma 5.2, we obtain that *A* still satisfies

$$\int_{0}^{+\infty }A(s)\;ds=1.$$

To conclude the existence proof of Theorem 5.1, we pass to the limit in the weak form of (43) as ε→0, R→∞.

Uniqueness is a standard property in Krein–Rutman theory and we refer to [18] for the particular example at hand.  □ 

### 5.2 The Nonlinear Case

**Theorem 5.4***Assume* (4)*–*(8), (12). *Then there is a steady solution*A(s)*to* (1).

*Proof* For a given N≥0, we know from the previous subsection that there is a solution A(s,N) to

$$\left\{ \begin{array}{c} \partial_{s}\bar{A}(s,N)+p(s,N)\bar{A}(s,N)=\int K(s,u)p(u,N)\bar{A}(u,N)\;du,\;s\geq 0,\\ \bar{A}(0,N)=0,\;\;\bar{A}(s,N)\geq 0,\;\;\int_{0}^{\infty }\bar{A}(s,N)\;ds=1. \end{array}\right.$$

A stationary state for the nonlinear equation is a fixed point to the mapping

$$N=F(N):=\int_{0}^{+\infty }p(s,N)\bar{A}(s,N)\;ds.$$

The following properties hold true by general continuity properties (that themselves follow from the bounds proved for the linear equation and the uniqueness of the solution to (42))

$$\begin{array}{rcl} & & F\text{ is continuous},\;\;F(0)>0,\\ & & F(N)\leq p_{M}\int_{0}^{+\infty }\bar{A}(s,N)\;ds=p_{M}. \end{array}$$

Therefore, there is at least one steady state. □

Notice that uniqueness is expected with the smallness assumptions (7) because $\left|F^{\prime }(N)\right|$ should be small (here, we leave this point without proof).

## 6 Numerical Simulations and Spontaneous Activity

When our smallness assumptions is not fulfilled, the neurons may undergo synchronization leading to a spontaneous activity of the network. The aim of this section is to illustrate this regime through numerical simulations and show the effect of the fragmentation term when the flux of neurons N(t) does not converge to a stationary state but oscillates.

To do so, we compare the dynamic of *N* in the two following “extreme” cases 

• $K(s,u)=\delta_{s=0}$ which is the case studied in [17] where all the neurons come back in a same state after discharge, 

• $K(s,u)=\delta_{s=u/2}$ when the neurons, after discharge, reach a state which is proportional to their time elapsed since discharge.

 Then we choose the discharge rate *p* as in the article [17], which allows us to obtain theoretically and numerically periodic solutions in the particular case where $K(s,u)=\delta_{s=0}$, that is,

(47) $$p\left(s,N(t)\right):=I_{s\geq \sigma (N(t))}$$

with a decreasing function *σ* built as follows: for α>0, we define the two functions

(48) $$0<N^{-}(\alpha ):=\frac{1}{2e^{\alpha }-1}<N^{+}(\alpha ):=\frac{e^{\alpha }}{2e^{\alpha }-1}<1,$$

and we choose the Lipschitz continuous discharge threshold *σ* as

(49) $$\sigma (x)=\{\begin{array}{cc} 2\alpha & \text{on }[0,N^{-}(\alpha )],\\ 2\alpha -\ln(x)+\ln(N^{-}(\alpha )) & \text{on }[N^{-}(\alpha ),N^{+}(\alpha )],\\ \alpha & \text{on }[N^{+}(\alpha ),\infty ). \end{array}$$

We now compare numerical simulations of the dynamic of *N* with the two kernels mentioned above. 

**Fig. 1:**
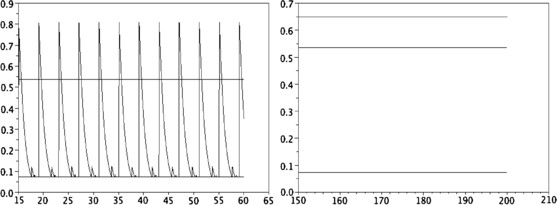
Total neural activity N(t) computed with α=2 and an initial data as $e^{-s}$. *Left*: $K(s,u)=\delta_{s=0}$. *Right*: $K(s,u)=\delta_{s=u/2}$. *The continuous lines* give the values $N_{-}$ and $N_{+}$ and in *the figure on the right*, N(t) is *the top line*

**Fig. 2:**
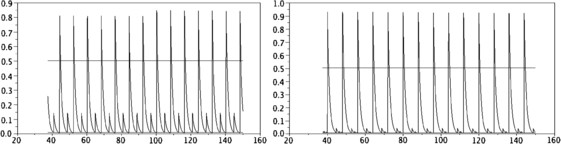
Total neural activity N(t) computed with α=4 and $K(s,u)=\delta_{s=0}$. *Left*: initial data as $e^{-s}$. *Right*: a more complex initial data (as in Proposition 2 of [17]). We see that two different periodic solutions occur with those two different initial data. *The continuous lines* give the values $N_{-}$ and $N_{+}$

**Fig. 3:**
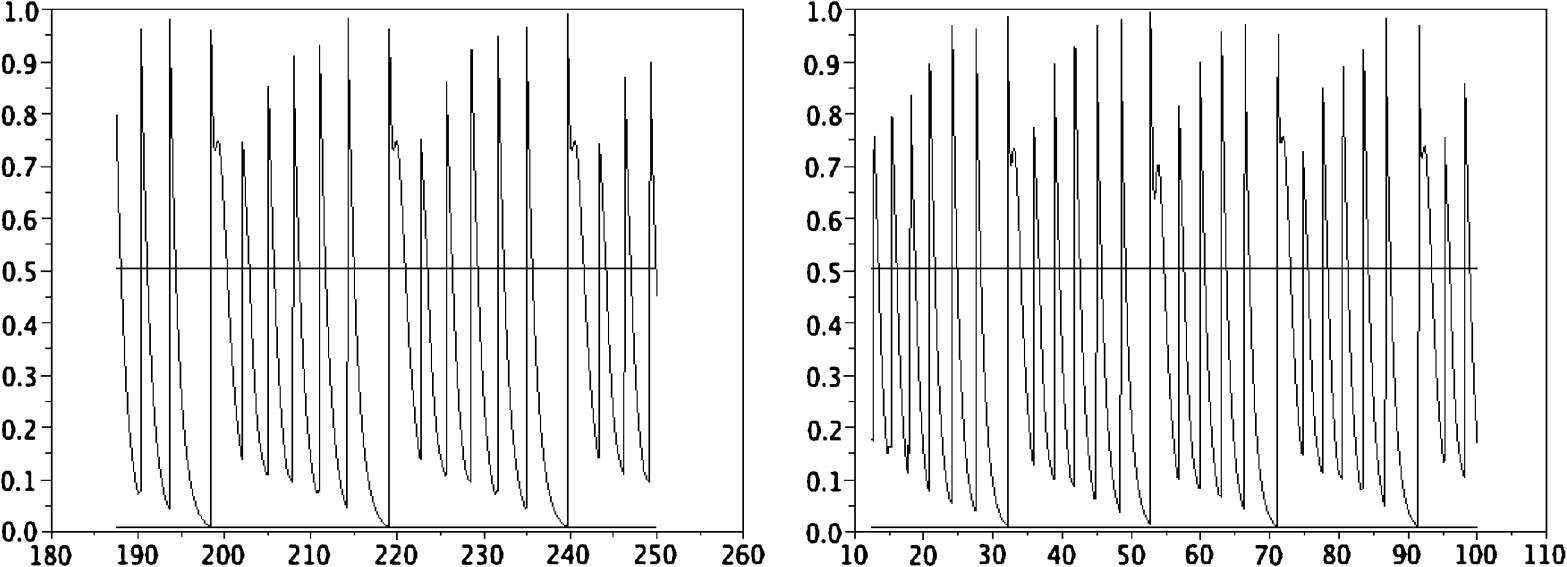
Total neural activity N(t) computed with α=4 and $K(s,u)=\delta_{s=u/2}$. *Left*: initial data as $e^{-s}$. *Right*: a more complex initial data (as in Proposition 2 of [17]). We observe that the two functions *N* are the same. *The continuous lines* give the values $N_{-}$ and $N_{+}$

**Fig. 4:**
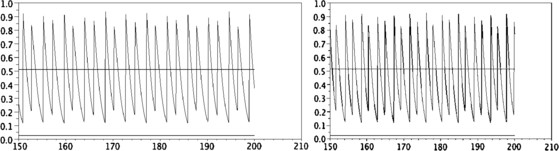
Total neural activity N(t) computed with α=3 and $K(s,u)=\delta_{s=u/2}$. *Left*: an initial data as $e^{-s}$. *Right*: a more complex initial data (as in Proposition 2 of [17]). We observe that the two functions *N* are the same. *The continuous lines* give the values $N_{-}$ and $N_{+}$

• For $K=\delta_{s=0}$, with this choice of function *p* given by (47), theoretical study and numerical results in [17] have shown that for all α>0, there exists a very large class of periodic solutions, Fig. 1 (left) depicts such a periodic solution. Moreover, with the numerical observations, the dynamic strongly depends on the initial data (see Fig. 2 and article [17]). 

• The kernel $K(s,u)=\delta_{s=u/2}$ seems to create a “smoothing effect.” Indeed, unlike the former case, when *α* is small enough, the numerical solution *N* converges to a stationary state (see Fig. 1, right). Moreover, for *α* fixed large enough, it seems that there does not exist a large spectrum of periodic solutions as in the case where $K(s,u)=\delta_{s=0}$; more precisely, we numerically obtain only one periodic solution (see Figs. 3 and 4). The numerical methods used here are analogous of those in article [17]; hence, we refer to this article for a description of the algorithm. 

## Appendix A: An Example of Coefficients *p* and *K*

The assumptions in Sect. 2.1 are abstract and we can explain their meaning for the particular case of the introduction that is, for two functions $\sigma :[0,\infty )\to [\sigma_{-},\sigma_{+}]$ and ψ:[0,∞)→[0,∞),

$$p(s,N)=H_{\delta }\left(s>\sigma (N)\right),\;\;K(s,u)=\delta \left(s-\psi (u)\right).$$

One can readily compute that

$$\begin{array}{rcl} f(s,u) & = & 1_{\left\{s>\psi (u)\right\}},\;\;\Phi (s,u)=\psi^{\prime }(u)\delta \left(s-\psi (u)\right),\\ \int_{0}^{\infty }\Phi (s,u)\;ds & = & \psi^{\prime }(u),\;\;\int_{0}^{u}sK(s,u)\;ds=\psi (u). \end{array}$$

Therefore, the assumption (12) is reduced to write

$$0\leq \psi^{\prime }(u)\leq \theta <1,\;\;\psi (u)\leq \theta u<u.$$

Finally the conditions (15) and (16) are reduced to saying that $\sigma_{+}$ and *θ* are small enough.

## Appendix B: A Direct Use of the Fundamental Formula on |M|

As mentioned earlier, in order to treat nonconstant coefficients, our fundamental new idea is to use the formulas (29), (30) on the integral M(s,t) which vanishes at s=0 and s=∞. We recall the later

(50) $$\begin{array}{rcl} & & \frac{\partial |M(s,t)|}{\partial t}+\frac{\partial |M(s,t)|}{\partial s}+p(s)\left|M(s,t)\right|\\ & & \;\leq \int_{u=s}^{\infty }\left[\left|p^{\prime }(u)\right|f(s,u)+p(u)\Phi (s,u)\right]\left|M(u,t)\right|\;du. \end{array}$$

We can give a direct proof of exponential decay for *M* based on this expression and using simpler assumptions on *p* and *K* that, however, do not cover the case when *p* can have large variation and p(s) can vanish for s≃0. It is a natural extension of the case when *p* is constant as covered in [12]. 

We use the definition, compatible with the example in Appendix A

$$\underline{\psi }(u)=\int_{0}^{u}f(s,u)\;ds=u-\psi (u),\;\;1-\theta \leq {\underline{\psi }}^{\prime }(u)\leq 1.$$

We are going to prove the following proposition.

**Proposition B.1***With the assumptions* (8), (10), (12), *if there exists*ν>0*such that*

$$-\left|p^{\prime }(u)\right|\underline{\psi }(u)+p(u){\underline{\psi }}^{\prime }(u)>\nu ,$$

then one has

$$\int_{0}^{\infty }\left|M(s,t)\right|\;ds\leq \int_{0}^{\infty }\left|M^{0}(s)\right|\;dse^{-\nu t}.$$

*Proof* We may integrate Eq. (50) and write, using the Fubini theorem

$$\begin{array}{rcl} & & \frac{d}{dt}\int_{0}^{\infty }\left|M(s,t)\right|\;ds+\int_{0}^{\infty }p(s)\left|M(s,t)\right|\;ds\\ & & \;\leq \int_{0}^{\infty }\int_{0}^{\infty }1_{\left\{0\leq s\leq u\right\}}\left[\left|p^{\prime }(u)\right|f(s,u)-p(u)\frac{\partial }{\partial u}f(s,u)\right]\left|M(u,t)\right|\;du\;ds\\ & & \;\leq \int_{0}^{\infty }\left[\left|p^{\prime }(u)\right|\underline{\psi }(u)-p(u){\underline{\psi }}^{\prime }(u)+p(u)\right]\left|M(u,t)\right|\;du. \end{array}$$

Therefore, we arrive at

$$\begin{array}{rcl} \frac{d}{dt}\int_{0}^{\infty }\left|M(s,t)\right|\;ds & = & \int_{0}^{\infty }\left[\left|p^{\prime }(u)\right|\underline{\psi }(u)-p(u){\underline{\psi }}^{\prime }(u)\right]\left|M(u,t)\right|\;du\\ & \leq & -\nu \int_{0}^{\infty }\left|M(u,t)\right|\;du. \end{array}$$

 □

## Appendix C: A Noncompact Eigenproblem

The proof of convergence to a steady state with an exponential decay rate relies on the existence of an eigenpair (λ<0, P>0) solution to

(51) $$-\frac{\partial P(s)}{\partial s}+\left(\lambda +p_{*}(s)\right)P(s)=\int_{0}^{s}\left[\left|p_{*}^{\prime }(s)\right|f(u,s)+p_{*}(s)\Phi (u,s)\right]P(u)\;du.$$

Its solution uses in a fundamental way the smallness assumptions of Sect. 2.1 linking *p* and *K*. Indeed, the following lemma holds.

**Lemma C.1***With the assumptions* (4), (5), (8), (10), (12), (15)*—on*$p_{*}(s)=p(s,A^{*})$*rather thanp—and onKfor*λ<0*close enough to* 0 *there is a unique solutionPto Eq*. (51) *with*P(0)=1*and there exist a constant*B>0*such that*

$$\frac{1}{B}\leq P(x)\leq BP(y)\;\text{for }0\leq x\leq y.$$

*Proof* Equation (51) is a delay differential equation, and thus has a global solution, i.e., for s≥0, for all *λ*. We consider a time interval $\left[0,s_{0}\right)$ where the solution *P* is positive and we prove that for λ>0 close enough to zero we can take $s_{0}=\infty$. We argue for λ=0 and then by continuity for *λ* close enough to 0.

The solution to Eq. (51) with λ=0 satisfies

(52) $$P(s)\leq e^{\int_{0}^{s}p_{*}(u)\;du}\;\text{for }0\leq s\leq s_{0}.$$

Therefore, integrating (51), we have for $s\leq \min(s_{0},\sigma^{*})$,

(53) $$P(s)\geq 1-e^{\int_{0}^{s}p_{*}(u)\;du}\left(\sigma^{*}\int_{0}^{s}\left|p_{*}^{\prime }(u)\right|\;du+\theta \int_{0}^{s}p_{*}(u)\;du\right)\geq 1-B_{*}>0$$

thanks to assumption (15). In particular, we conclude that $s_{0}>\sigma^{*}$.

At this stage, we may argue by continuity and for λ>0 close enough to 0, we still have

(54) $$\begin{array}{rl} P(s) & \leq e^{\int_{0}^{s}p_{*}(u)\;du}+O(\lambda ),\\ P(s) & \geq 1-B_{*}+O(\lambda )>0\;\text{for }0\leq s\leq \sigma^{*}. \end{array}$$

For $s>\sigma^{*}$, we write (51) as

$$P^{\prime }(s)=P(s)\left[p_{M}+\lambda -\int_{0}^{s}\Phi (u,s)\frac{P(u)}{P(s)}\;du\right].$$

We are going to prove that *P* is increasing for $s>\sigma^{*}$, that is the bracket is positive. This is because we can write

$$\int_{0}^{s}\Phi (u,s)\frac{P(u)}{P(s)}\;du\leq \int_{0}^{\sigma^{*}}\Phi (u,s)\;du\frac{{∥P∥}_{L^{\infty }(0,\sigma^{*})}}{\inf_{s\in (0,\sigma^{*})}P(s)}+\int_{\sigma^{*}}^{s}\Phi (u,s)\;du.$$

Estimates (54) on *P* and assumption (15), tell us that

$$\int_{0}^{\sigma^{*}}\Phi (u,s)\;du\frac{{∥P∥}_{L^{\infty }(0,\sigma^{*})}}{\inf_{s\in (0,\sigma^{*})}P(s)}+\int_{\sigma^{*}}^{s}\Phi (u,s)\;du\leq p_{M}.$$

Therefore, we obtain that for λ<0 close enough to 0 then the bracket is positive and *P* is increasing on $\left[\sigma^{*},+\infty \right)$ which proves Lemma C.1. □

## Appendix D: A Consequence of the Krein–Rutman Theorem

To prove the existence of steady states, we have used the existence of a solution to a regularized eigenfunction problem. Namely, we have the

**Theorem D.1** ([7,18]) 

*Let*R>0, $E=C^{0}([0,R])$*and let*

$$0\leq B(\cdot )\in E,\;\;0\leq b(\cdot ,\cdot )\in C^{0}\left([0,R]\times [0,R]\right),$$

*and let*ε>0*be small enough*. *Then there is a unique*λ∈R, $A\in C^{1}([0,R])$, *solution of the equation*

$$\left\{ \begin{array}{c} \frac{\partial A(s)}{\partial s}+(B(s)+\lambda )A(s)=\int_{0}^{R}b(s,u)A(u)\;du,\;0\leq s\leq R,\\ A(0)=\varepsilon \int_{0}^{R}A(y)\;dy,\;\;A(s)>0,\;\;\int_{0}^{R}A(s)\;ds=1. \end{array}\right.$$

## Competing Interests

The authors declare that they have no competing interests.

## Authors’ Contributions

The authors have equally contributed to this paper.

## References

[B1] Balagué D, Cañizo JA, Gabriel P: **Fine asymptotics of profiles and relaxation to equilibrium for growth-fragmentation equations with variable drift rates**. Preprint; 2012.

[B2] Bansaye V Tran VC Branching Feller diffusion for cell division with parasite infection ALEA Lat Am J Probab Math Stat 2011 4 95 127

[B3] Bardet J-B, Christen A, Guillin A, Malrieu F, Zitt P-A: **Total variation estimates for the TCP process**. Preprint; 2011.

[B4] Cáceres MJ Cañizo JA Mischler S Rate of convergence to self-similarity for the fragmentation equation in $L^{1}$ spaces Commun Appl Ind Math 2011 4 2 299 308

[B5] Cáceres MJ Cañizo JA Mischler S Rate of convergence to an asymptotic profile for the self-similar fragmentation and growth-fragmentation equations J Math Pures Appl 2011 4 4 334 362 10.1016/j.matpur.2011.01.003

[B6] Calvez V Lenuzza N Doumic M Deslys J-P Mouthon F Perthame B Prion dynamic with size dependency—strain phenomena J Biol Dyn 2010 4 1 28 42 10.1080/17513750902935208 22881069

[B7] Dautray R Lions J-L Mathematical Analysis and Numerical Methods for Sciences and Technology 1990 Springer, Berlin

[B8] Doumic Jauffret M Gabriel P Eigenelements of a general aggregation-fragmentation model Math Models Methods Appl Sci 2010 4 5 757 783 10.1142/S021820251000443X

[B9] Engler H Pruss J Webb GF Analysis of a model for the dynamics of prions II J Math Anal Appl 2006 4 98 117 10.1016/j.jmaa.2005.11.021

[B10] Farkas JZ Hagen T Stability and regularity results for a size-structured population model J Math Anal Appl 2007 4 1 119 136 10.1016/j.jmaa.2006.05.032

[B11] Gabriel P: **Long-time asymptotics for nonlinear growth-fragmentation equations**. arXiv:1102.2871; 2011. 1102.2871

[B12] Laurençot P Perthame B Exponential decay for the growth-fragmentation/cell-division equations Commun Math Sci 2009 4 2 503 510 10.4310/CMS.2009.v7.n2.a12

[B13] Laurençot P Walker C Well-posedness for a model of prion proliferation dynamics J Evol Equ 2007 4 241 264 10.1007/s00028-006-0279-2

[B14] Michel P Existence of a solution to the cell division eigenproblem Math Models Methods Appl Sci 2006 4 7 1125 1153

[B15] Michel P Mischler S Perthame B General relative entropy inequality: an illustration on growth models J Math Pures Appl 2005 4 9 1235 1260 10.1016/j.matpur.2005.04.001

[B16] Pakdaman K Perthame B Salort D Dynamics of a structured neuron population Nonlinearity 2010 4 55 75 10.1088/0951-7715/23/1/003

[B17] Pakdaman K Perthame B Salort D Relaxation and self-sustained oscillations in the time elapsed neuron network model SIAM J Appl Math 2013 4 3 1260 1279 10.1137/110847962

[B18] Perthame B Transport Equations in Biology Frontiers in Mathematics 2007 Birkhäuser, Basel

[B19] Perthame B Ryzhik L Exponential decay for the fragmentation or cell-division equation J Differ Equ 2005 4 155 177 10.1016/j.jde.2004.10.018

[B20] Simonett G Walker C On the solvability of a mathematical model for prion proliferation J Math Anal Appl 2006 4 1 580 603 10.1016/j.jmaa.2005.12.036

